# Chemotherapeutic perfusion of portal vein after tumor thrombectomy and hepatectomy benefits patients with advanced hepatocellular carcinoma: A propensity score‐matched survival analysis

**DOI:** 10.1002/cam4.2556

**Published:** 2019-09-30

**Authors:** Yang Gao, Peng‐Xiang Wang, Jian‐Wen Cheng, Yun‐Fan Sun, Bo Hu, Wei Guo, Kai‐Qian Zhou, Yue Yin, Yuan‐Cheng Li, Jian Wang, Jun‐Feng Huang, Shuang‐Jian Qiu, Jian Zhou, Jia Fan, Xin‐Rong Yang

**Affiliations:** ^1^ Department of Liver Surgery & Transplantation Liver Cancer Institute Zhongshan Hospital Fudan University Shanghai P. R. China; ^2^ Key Laboratory of Carcinogenesis and Cancer Invasion Ministry of Education Shanghai P. R. China; ^3^ Department of Laboratory Medicine Zhongshan Hospital Fudan University Shanghai P. R. China; ^4^ Institutes of Biomedical Sciences Fudan University Shanghai China; ^5^ Department of Intensive Care Medicine Zhongshan Hospital Fudan University Shanghai China

**Keywords:** hepatocellular carcinoma, portal vein chemotherapy, portal vein tumor thrombosis, recurrence, sorafenib

## Abstract

**Background:**

Portal vein tumor thrombus (PVTT) is a common complication in hepatocellular carcinoma (HCC), signaling dismal outcomes. This study was conducted to evaluate the survival benefit of postoperative portal vein perfusion chemotherapy (PVC) in patients with HCC and PVTT.

**Methods:**

A retrospective review was conducted in 401 consecutive patients with HCC and PVTT who underwent hepatic resection between January 2009 and December 2015 and 67 patients received adjuvant postoperative PVC. A propensity score matching (PSM) was used to match patients with and without PVC at a ratio of 1:1.

**Results:**

After PSM, the median time to recurrence (TTR) and overall survival (OS) were significantly longer in PVC group compared with control group (12.3 vs 5.8 months, *P* = .001; 19.0 vs 13.4 months, *P* = .037; respectively). At 1, 2, 3, and 5 years, the cumulative recurrence rates in PVC group were 48.1%, 86.5%, 92.3% ,96.2%, respectively, with OS rates of 63.8%, 37.9%, 24.4%, 18.3%, respectively; whereas cumulative recurrence rates of 76.6%, 91.5%, 94.3%, and 97.2%, respectively and OS rates of 55.4%, 23.0%, 12.4%, and 12.4%, respectively were recorded for the control group. In multivariate analysis, postoperative PVC emerged as a significant predictor for TTR (hazard ratio [HR], 0.523; *P* = .001) and OS (HR, 0.591; *P* = .010). PVC could reduce early recurrence (≤1 year) rate after surgical resection (40.3% vs 64.2%, *P* = .006) and clinical outcomes were further enhanced by adding sorafenib to postoperative PVC.

**Conclusions:**

Compared with surgical resection alone, postoperative adjuvant PVC treatment boosts survival and reduces early tumor recurrences in patients surgically treated for HCC and PVTT.

## Graphical abstract

Surgical resection is an option for patients with HCC and PVTT and multidiscipline treatment including portal vein chemotherapy may further improve clinical outcomes. Patients treated with and without postoperative PVC were selected by propensity score matching analysis (PSM) to eliminate potential confounder factors. Before PSM, there was no significant difference between time to recurrence (TTR) and overall survival (OS). After PSM, patients receiving postoperative PVC had significant longer TTR and OS.

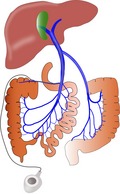

## 1 INTRODUCTION

Hepatocellular carcinoma (HCC) is the most common primary liver cancer, ranking fourth in the 2018 projections of cancer‐related deaths worldwide.^1^ There is a proclivity for vascular invasion within and around the liver, including the portal, hepatic, or superior mesenteric vein, and even inferior vena cava;^2^ but portal vein tumor thrombosis (PVTT) is the most frequent event. Macroscopic PVTT is evident in about 10%‐40% of patients at the time of diagnosis, and median survival time (MST)  of this subpopulation of patients was 2.7‐4.0 months if left untreated.^3^, ^4^ The Barcelona Clinic Liver Cancer staging system classifies such patients as advanced disease (stage C) and advocates sorafenib as standard therapy, although the MST of advanced HCC patients given sorafenib is only 10.7 months.^5^, ^6^ Surgical resection may therefore have merit in certain patients, offering a chance for long‐term survival.^7^ In China, the 2017 guidelines for diagnosis and treatment of primary liver cancer do recommend surgical intervention for some patients with PVTT.^8^ However, high incidence of postoperative tumor recurrence in HCC patients with PVTT limited the efficacy of surgical treatment,^6^ and multimodality therapeutic strategies should be considered to prolong postoperative survival of those patients.

Portal vein perfusion chemotherapy (PVC) may be used postoperatively in patients with HCC complicated by PVTT to concentrate local drug delivery and reduce systemic side effects.^9^ However, few studies to date have addressed the clinical efficacy of PVC in this setting.^10^, ^11^, ^12^ Past studies of ours underscore the promise of continuous postoperative PVC in these scenarios, showing prolonged survival and fewer recurrences,^13^, ^14^, ^15^ but this approach to HCC remains controversial and is not routinely applied in clinical practice. A prospective study of patients with HCC and PVTT to explore its clinical efficacy would nevertheless prove challenging. Propensity score matching (PSM) instead serves to overcome selection bias, offset differing clinical features among groups, and bolsters the evidence level of a retrospective observational study,^16^, ^17^ providing a workable means of investigation.

This retrospective study aimed to specially investigate whether HCC patients with PVTT would benefit from PVC after hepatectomy and tumor thrombectomy. PSM analysis was applied to minimize the potential confounding factors to facilitate more reliable conclusion. In this study, 510 patients who underwent resections of HCC and PVTT at our institute in the past 7 years were enrolled, assigning consecutive patients to PVC (n = 67) and control (n = 67) groups by PSM analysis. We then compared the clinical features and prognoses of these two groups after surgery, assessing the efficacy of postoperative PVC treatment in patients with HCC and PVTT. Factors impacting disease recurrence and survival were also evaluated. We found that postoperative PVC could prolong overall survival of HCC patients with PVTT through reducing early recurrence, which paved an alternative way for improving clinical outcomes in HCC patients with PVTT.

## 2 PATIENTS AND METHODS

### 2.1 Patients

**Figure 1:**
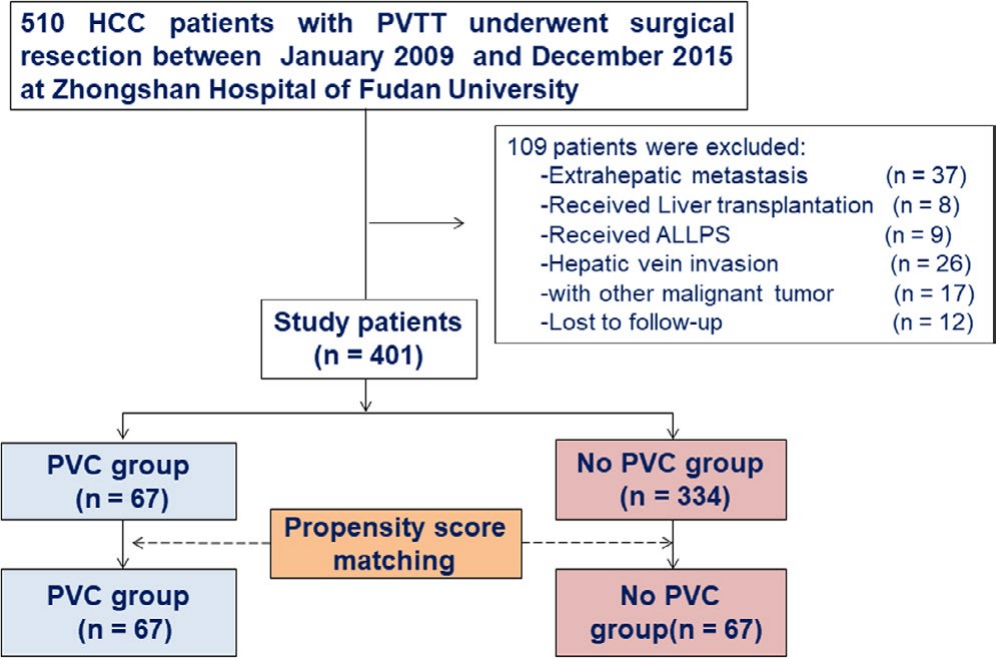
Flowchart of patient selection for the study. ALLPS, associating liver partition and portal vein ligation for staged hepatectomy; HCC, hepatocellular carcinoma; PVC, portal vein chemotherapy; PVTT, portal vein tumor thrombus.

From January 2009 to December 2015, 510 consecutive patients who underwent surgery for HCC with PVTT at Zhongshan Hospital of Fudan University were retrospectively reviewed and enrolled in the study. Inclusion criteria in this study were as follows: (a) HCC with macroscopic tumor thrombus involving segmental branches of the portal vein or above (type I), thrombus in the tumor thrombi extend to include the right/left portal vein (type II) or the main portal vein is involved (type III), which was confirmed by preoperative diagnoses or intraoperative exploration;^18^ (b) resectable primary tumor, and the PVTT can be removed together with the tumors; (c) good or moderate hepatic function (Child‐Pugh A or B); (d) no tumor invasion in hepatic arteries, bile ducts or inferior vena cava; (e) absence of extrahepatic metastasis; (f) no prior anticancer treatment and (g) no contraindications to laparotomy. Exclusion criteria were as follows: (a) liver function of Child‐Pugh C; (b) received ALLPS (associated liver partition and portal vein ligation for staged hepatectomy) or liver transplantation; (c) coexistence of other malignancies; (d) incomplete follow‐up data. As a result, 109 patients failed to meet the inclusion criteria and were excluded in this study and 401 patients were enrolled, including 67 patients with postoperative adjuvant PVC (PVC group) and 334 patients without PVC (control group) (Figure 1). HCC was diagnosed histologically by examining surgically resected specimens. Tumor differentiation was graded according to the Edmondson grading system.^19^ PVTT type was classified according to Cheng's classification system.^18^ Liver function was assessed using the Child‐Pugh scoring system^20^ and tumor stage was determined in accordance with the BCLC (Barcelona Clinic Liver Cancer) staging system^6^ or Chinese HCC staging system.^8^

The study protocol was approved by the Institutional Ethics Committee of the Zhongshan Hospital, Fudan University on 27/2/2018 and was conducted according to the ethics guidelines of 1975 Declaration of Helsinki. Written informed consent was granted by each of the recruited patients.

### 2.2 Surgery, PVC procedures and other adjuvant treatments

**Figure 2:**
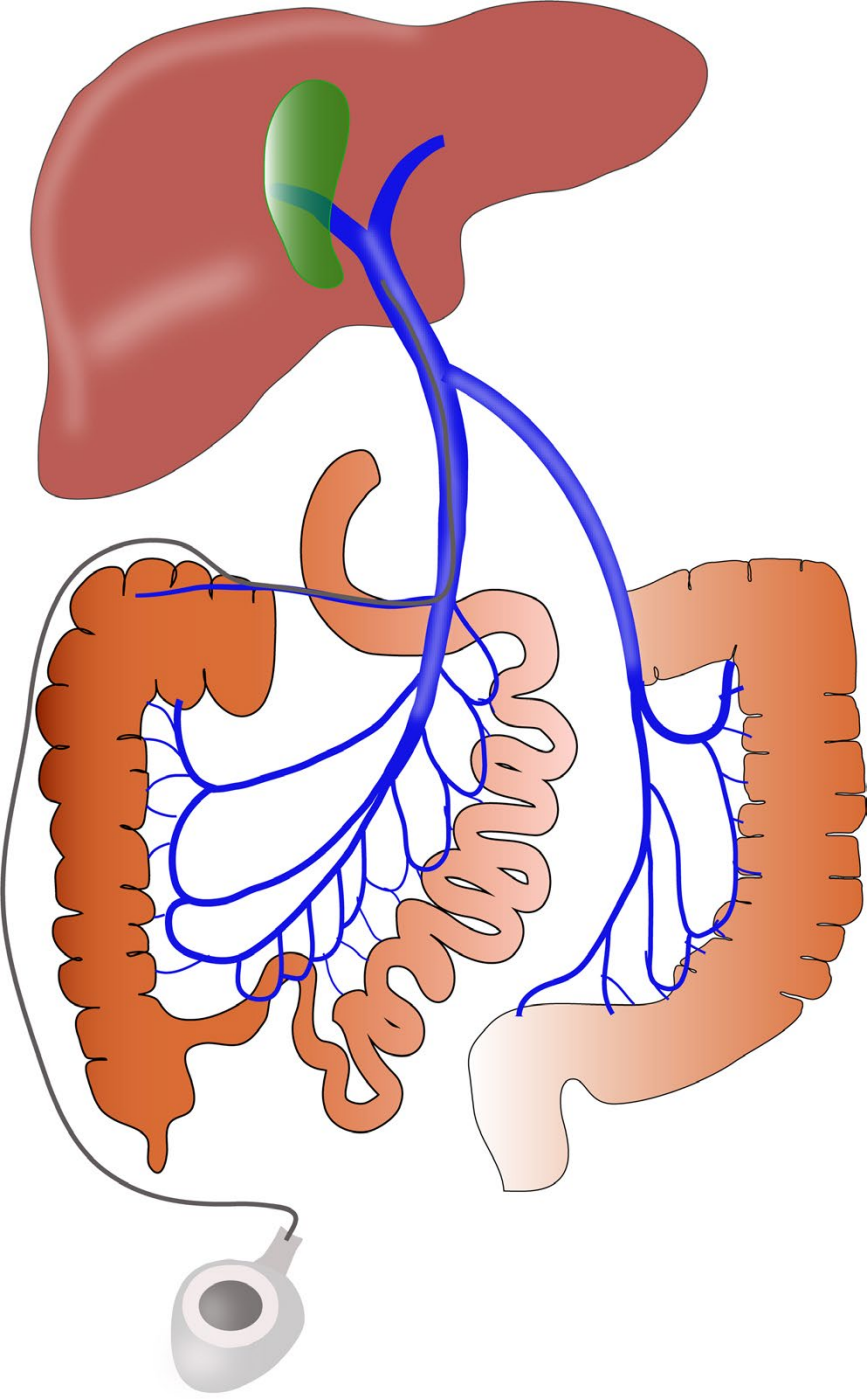
Positioning of a portal vein infusion pump

Based on the location, size, and number of tumors, various types of hepatectomy and thrombectomy were performed in HCC patients with PVTT. Tumor thrombi were extracted or excised prior to hepatectomy in all patients. In the PVC group, the catheter of the infusion pump was cannulated into the portal trunk via the right gastroepiploic vein or middle colic vein, and anticancer drugs were delivered postoperatively by a continuous pump via a subcutaneously implanted injection port by a continuous infusion pump as our previous report (Figure 2).^13^ To prevent the catheter from occluding, injection ports were flushed daily for 1 week after surgery with 20 mL (100 U/mL) of low‐molecular‐weight heparin solution. Chemotherapy was initiated 1‐2 weeks after surgery when the hepatic function basically recovered to the normal level, the chemotherapy regimen was as follows: cisplatin (40 mg/m^2^/d) and doxorubicin (30 mg/m^2^/d) at day 1, 5‐FU (650 mg/m^2^/d) on day 1 and 2. The regimen was repeated every 4 weeks, and the number of chemotherapy courses varied from 2 to 7 according to the patient's tolerance. Liver function, blood cell count, and renal function were monitored during each course. If the patients had multiple and larger tumors (>10 cm), they were advised to receive TACE or sorafenib treatment. However, the final postoperative treatment depended on the patient's socioeconomic status and compliance with doctors.

### 2.3 Follow‐up

Postoperative patient surveillance was performed as described previously.^21^ In brief, all patients were followed every 2 months within postoperative 1 year and once every 3 to 4 months thereafter. Every patient was prospectively monitored by liver function test, serum alpha‐fetoprotein (AFP), hematological parameters and abdomen ultrasonography every 1 to 6 months during the postoperative period. Magnetic resonance imaging (MRI) and/or computed tomography (CT) of the abdomen was performed every 6 months. If intrahepatic recurrence or distal metastasis was clinically suspected on the basis of symptoms or unexplained elevation of tumor marker levels, MRI, CT or bone scan was performed immediately. If recurrence occurred during the follow‐up, optimal treatment of the recurrent tumor was chosen depending on tumor location, size, number of lesions and liver function.

Follow‐up was terminated on 31 December 2017. Time to recurrence (TTR) was defined as the interval from the date of resection to date of the first documented tumor recurrence, death, or the last follow‐up visit. Overall survival (OS) was defined as the interval from surgery to date of death or the last follow‐up visit. We used 12 months after surgical resection as the cutoff value to divide all recurrences into early recurrence (≤1 year) and late recurrence (>1 year).^21^

### 2.4 Propensity score matching analysis

PSM analysis was conducted to overcome potential selection bias^22^ in our cohort arising from the fact that patients were not randomized into PVC group or control group. Variables showing statistically significant differences (*P* < .05) were included in the PSM analysis. Specifically, we calculated the propensity scores (from 0 to 1) using the logistic regression model and matched at 1:1 ratio to balance the baseline differences of the patients who underwent surgical resection only and those receiving postoperative PVC. Covariates employed for propensity score model included: gender (male/female), age (≤50/>50 years), degree of PVTT (type I‐II/III), Edmondson stage (I‐II/III‐IV), tumor number(single/multiple), tumor encapsulation (none/complete), tumor size (≤5/>5 cm), AFP (≤400/>400 ng/mL), hepatitis C virus (HCV) infection, hepatitis B surface antigen (HBsAg) (negative/positive), HBV DNA (≤1 × 10^4^/>1 × 10^4^ IU/mL), alanine aminotransferase (ALT) (≤75/>75 U/L), gamma glutamyl transpeptidase (GGT) (≤54/>54 U/L), albumin (≤3.5/>3.5 g/dL), platelet count (≤100 × 10^9^/>100 × 10^9^/L), prothrombin time (PT) (≤13/>13 seconds), liver cirrhosis (No/Yes), and Child–Pugh class (A/B). Matches were generated one‐to‐one without replacement between PVC and control group member using the nearest‐neighbor matching algorithm and a 0.05 caliper value. Sixty‐seven matched pairs were generated for subsequent analyses.

### 2.5 Statistical analysis

Demographics, clinical features and tumor characteristics of the study population are described as mean (± standard deviation), median (range), or percentage according to nature of the data. Continuous variables were compared by group using Student's *t* test or the Wilcoxon rank sum test. Categorical data were analyzed via *χ* ^2^ or Fisher's exact test. Cumulative recurrence and survival rates were estimated using the Kaplan‐Meier method applying log‐rank test. Univariate and multivariate analysis were performed using the Cox proportional hazards regression model. The prognostic factors with *P* < .05 in univariate analysis were subjected to the final multivariate analysis.

Two‐tailed *P* < .05 was considered statistically significant. The PSM analysis was carried out using R version 3.3.2 (R Foundation for Statistical Computing, Vienna, Austria). Statistical analysis was performed using GraphPad Prism 6.0 (GraphPad Software, San Diego, CA).

## 3 RESULTS

### 3.1 Baseline characteristics of patients with HCC and PVTT after operation

Baseline clinicopathological characteristics of all patients (n = 401) are summarized in Table 1. Only 67 patients (16.7%) received postoperative PVC and the other 344 patients (83.3%) did not. Compared to control group, patients in PVC group exhibited higher percentage of young patients (<50 years), more advanced degree of PVTT, larger tumor size and higher ALT levels. There were no significant differences in sex, Edmondson stage, tumor number, tumor encapsulation, AFP level, HBsAg, HBV DNA, ALT, GGT, platelet, liver cirrhosis and Child‐Pugh class (Table 1). Overall, the median follow‐up period was 12.3 months (range, 0.3‐105.4 months). In the control group, 214 patients underwent TACE, 36 patients received sorafenib therapy and 151 patients did not receive either TACE or sorafenib but may receive other treatments, such as traditional Chinese medicine or antiviral therapy after operation. For 67 patients receiving postoperative PVC, 29 received TACE, eight received sorafenib and the other 30 patients did not receive either one.

**Table 1:** Clinicopathologic characteristics of patients in PVC group and control group before and after propensity score matching

Variable	Before propensity score matching			After propensity score matching		
	PVC	Control	*P* value	PVC	Control	*P* value
Sex			.652^a^			.561^a^
Female	5 (7.5%)	33 (9.9%)		5 (7.5%)	8 (11.9%)	
Male	62 (92.5%)	301 (90.1%)		62 (92.5%)	59 (88.1%)	
Age (y)			**.033**			.861
≤50	39 (58.2%)	147 (44.0%)		39 (58.2%)	40 (59.7%)	
>50	28 (41.8%)	187 (56.0%)		28 (41.8%)	27 (40.3%)	
Degree of PVTT			**<.001** a			.706
Type Ⅰ‐Ⅱ	46(68.7%)	302 (90.4%)		46 (68.7%)	48 (71.6%)	
Type Ⅲ	21 (31.3%)	32 (9.6%)		21 (31.3%)	19 (28.4%)	
Edmondson stage			.663			.213
Ⅰ‐Ⅱ	29 (43.3%)	135 (40.4%)		29 (43.3%)	22 (32.8%)	
Ⅲ‐Ⅳ	38 (56.7%)	199 (59.6%)		38 (56.7%)	45 (67.2%)	
Tumor number			.704			.117
Single	34 (50.7%)	161 (48.2%)		34 (50.7%)	25 (37.3%)	
Multiple	33 (49.3%)	173 (51.8%)		33 (49.3%)	42 (62.7%)	
Tumor encapsulation			.851^a^			.585^a^
None	58 (86.6%)	284 (85.0%)		58 (86.6%)	61 (91.0%)	
Complete	9 (13.4%)	50 (15.0%)		9 (13.4%)	6 (9.0%)	
Tumor size(cm)			**.015**			1.000
≤5	10 (14.9%)	98 (29.3%)		10 (14.9%)	10 (14.9%)	
>5	57 (85.1%)	236 (70.7%)		57 (85.1%)	57 (85.1%)	
AFP(ng/mL)			.908			.484
≤400	30 (44.8%)	147 (44.0%)		30 (44.8%)	26 (38.8%)	
>400	37 (55.2%)	187 (56.0%)		37 (55.2%)	41 (61.2%)	
Anti‐HCV			1.000			.511
Negative	63 (94.0%)	313 (93.7%)		61 (91.0%)	63 (94.0%)	
Positive	4 (6.0%)	21 (6.3%)		6 (9.0%)	4 (6.0%)	
HBsAg			.688^a^			.791^a^
Negative	7 (10.4%)	43 (12.9%)		7 (10.4%)	9 (13.4%)	
Positive	60 (89.6%)	291 (87.1%)		60 (89.6%)	58 (86.6%)	
HBV DNA (IU/mL)			.873			.579
≤10^4^	47 (70.1%)	231 (69.2%)		47 (70.1%)	44 (65.7%)	
>10^4^	20 (29.9%)	103 (30.8%)		20 (29.9%)	23 (34.3%)	
ALT(U/L)			**.029**			.812
≤75	56 (83.6%)	310 (92.8%)		56 (61.2%)	57 (59.7%)	
>75	11 (16.4%)	24 (7.2%)		11 (38.8%)	10 (40.3%)	
GGT(U/L)			.205			.476
≤54	12 (17.9%)	84 (25.1%)		12 (17.9%)	9 (28.4%)	
>54	55 (82.1%)	250 (74.9%)		55 (82.1%)	58 (71.6%)	
Albumin (g/dL)			.419			.511
≤3.5	4 (6.0%)	30(9.0%)		6 (9.0%)	4 (6.0%)	
>3.5	63 (94.0%)	304 (91.0%)		61 (91.0%)	63 (94.0%)	
PLT (×10^9^/L)			.743			1.000
≤100	14 (20.9%)	64 (19.2%)		14 (20.9%)	14 (20.9%)	
>100	53 (79.1%)	270 (80.8%)		53 (79.1%)	53 (79.1%)	
PT(s)			.252			.612
≤13	59 (88.1%)	275 (82.3%)		59 (88.1%)	57 (88.1%)	
>13	8(11.9%)	59 (17.7%)		8 (14.9%)	10 (11.9%)	
Liver cirrhosis			.984			.861
No	29 (43.3%)	145 (43.4%)		29 (43.3%)	28 (41.8%)	
Yes	38 (56.7%)	189 (56.6%)		38 (56.7%)	39 (58.2%)	
Child–Pugh class			.781^a^			.718
A	64 (95.5%)	312 (93.4%)		64 (95.5%)	62 (92.5%)	
B	3 (4.5%)	22 (6.6%)		3 (4.5%)	5 (7.5%)	

Note

Significant *P*‐values are shown in bold.

Abbreviations: AFP, alpha‐fetoprotein; ALT, alanine aminotransferase; GGT, gamma glutamyl transpeptidase; HBsAg, hepatitis B surface antigen; PLT, platelet count; PVC, portal vein chemotherapy; PVTT, portal vein tumor thrombosis.

a Fisher's exact test.

Before PSM, univariate analyses in HCC patients with PVTT indicated that tumor size (HR, 1.561; 95% CI, 1.227‐1.985; *P* < .001) and liver cirrhosis (HR, 1.347; 95% CI, 1.078‐1.682; *P* = .009) were independently associated with tumor recurrence; tumor size (HR, 2.445; 95% CI, 1.813‐3.298; *P* < .001), AFP level (HR, 1.599; 95%CI, 1.254‐2.038; *P* < .001), HBV DNA (HR, 1.526; 95%CI, 1.187‐1.962; *P* = .001), GGT (HR, 1.573; 95% CI: 1.171‐2.114; *P* = .003) and PT (HR, 1.424; 95%CI, 1.048‐1.935; *P* = .024) were independently associated with overall survival. In multivariate analysis, tumor size (HR, 1.591; 95%CI, 1.250‐2.026; *P* **<**.001) and liver cirrhosis (HR, 1.381; 95% CI, 1.105‐1.726; *P* = .005) were independent predictive factors for TTR, while tumor size (HR, 2.206; 95% CI, 1.626‐2.994; *P* < .001) and AFP level (HR, 1.307; 95% CI, 1.018‐1.679; *P* = .036) were independent predictive factors for OS (Table S1).

### 3.2 The clinical efficacy of postoperative PVC in patients with HCC and PVTT before PSM analysis

**Figure 3:**
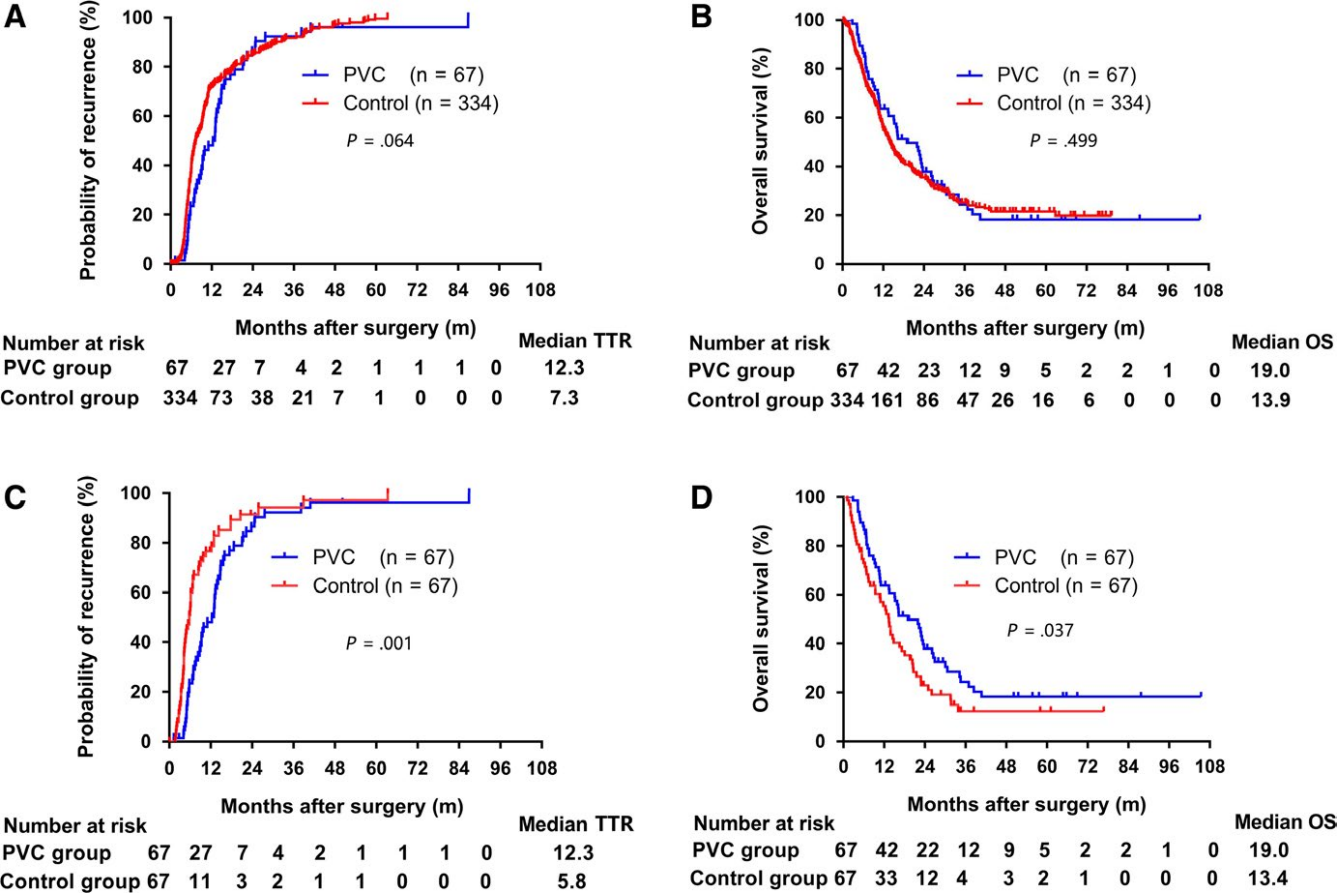
Kaplan‐Meier estimates for time to recurrence (TTR) and overall survival (OS) according to treatment type. Cumulative recurrence rate (A) and overall survival rate (B) in patients with HCC and PVTT in PVC group and control group before propensity score matching. Cumulative recurrence rate (C) and overall survival (D) in patients with HCC and PVTT in PVC group and control group after propensity score matching. Numbers below the x‐axis indicate the number of patients at risk. The log‐rank test was used for comparison

At the time of last follow‐up, 50 of 67 (74.6%) patients in PVC group and 227 of 334 (70.0%) patients in control group were deceased, 53 of 67 (79.1%) patients in PVC group and 271 of 334 (81.1%) patients in control group experienced postoperative recurrence. Before PSM, there were no significant differences in postoperative TTR and OS between PVC group and control group (12.3 vs 7.3 months, *P* = .064; 19.0 vs 13.9 months, *P* = .499; respectively). The 1‐, 2‐, 3‐, and 5‐year cumulative recurrence rates were 48.1%, 86.5%, 92.3%, 96.2% in PVC group and 71.9%, 85.0%, 91.7%, 99.6% in the control group, respectively (Figure 3A). The overall cumulative 1‐, 2‐, 3‐, and 5‐year OS rates were 63.8%, 37.9%, 24.4%, 18.3% in the PVC group and 55.2%, 35.6%, 25.2%, 21.5% in the control group, respectively (Figure 3B).

### 3.3 Clinical efficacy of postoperative PVC in patients with HCC and PVTT after PSM analysis

A total of 67 pairs of patients in PVC group and control group were finally matched. The median follow‐up time was 13.5 months (range, 1.1‐105.4 months). Clinicopathological characteristics of study patients after propensity score matching are shown in Table 1. After PSM, there were no significant differences in variables between the PVC group and control group as presented in Table 1. After PSM, the median TTR and OS were both significantly longer in PVC group compared with control group (12.3 vs 5.8 months, *P* = .001; 19.0 vs 13.4 months, *P* = .037; respectively). The 1‐,2‐, 3‐, and 5‐year cumulative recurrence rates and OS rates were 48.1%, 86.5%, 92.3%, 96.2% and 63.8%, 37.9%, 24.4%, 18.3% in PVC group respectively. While the 1‐,2‐, 3‐, and 5‐year cumulative recurrence and OS rates were 76.6%, 91.5%, 94.3%, 97.2% and 55.4%, 23.0%, 12.4%, 12.4% in control group, respectively (Figure 3C,D).

### 3.4 Prognostic factors in patients with HCC and PVTT after PSM analysis

**Figure 4:**
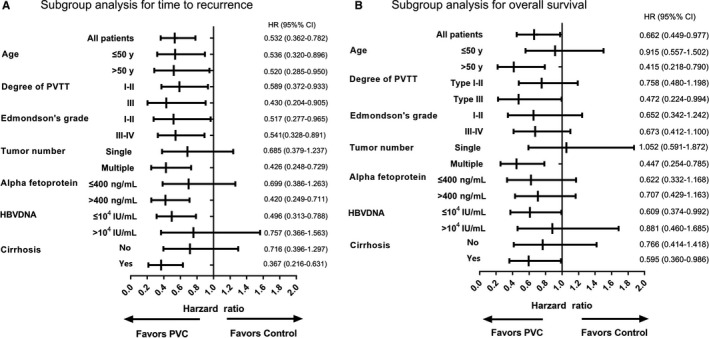
Subgroup analyses for time to recurrence (A), and overall survival (B) between PVC group and control group by Cox regression. PVTT, portal vein tumor thrombosis; HR, hazard ratio; CI, confidence interval

Since sex, age, degree of PVTT, Edmondson stage, tumor number, tumor encapsulation, tumor size, AFP level, HCV, HBsAg, HBV DNA, AST, GGT, albumin, platelet count, PT, degree of cirrhosis, and Child‐Pugh stage were potential prognostic factors for HCC patients, they were taken into consideration in the PSM analysis.^23^, ^24^ To further investigate the influence of the PVC treatment on HCC patients with PVTT after operation, the Cox proportional hazards model was used to analyze the prognostic factors of therapeutic outcomes of PVC. In univariate analysis, PVC, tumor size and GGT level were statistically significant clinical factors for both TTR and OS. In addition, univariate analysis also identified HBV DNA level, PT and liver cirrhosis were prognostic factors for OS. For TTR, postoperative PVC (HR, 0.532; 95% CI, 0.361‐0.784; *P* = .001) and tumor size (HR, 1.701; 95% CI, 1.001‐2.890; *P* = .049) emerged as independent prognostic factors by multivariate analysis (Table 2). For OS, multivariate analysis revealed that postoperative PVC (HR, 0.591; 95% CI, 0.395‐0.883; *P* = .010) and tumor size (HR, 3.555; 95% CI, 1.744‐7.245; *P* < .001) were independent prognostic factors (Table 2). Subgroup analysis was done based on various factors correlated with the prognosis (TTR and OS) of HCC patients with PVTT after resection, including age, degree of PVTT, Edmondson's grade, tumor number, AFP level, HBV DNA status, and liver cirrhosis. The HRs of the majority of these analyses were less than one, indicating that postoperative PVC tended to confer potential clinical benefit in most of the exploratory subgroups (Figure 4).

**Table 2:** Univariate and multivariate Cox regression analyses of clinicopathologic characteristics in HCC patients with PVTT after PSM

Variable	TTR		OS	
	HR (95% CI)	*P* value	HR (95% CI)	*P* value
Univariate analysis				
Sex (male vs female)	0.975 (0.520‐1.829)	.938	1.267 (0.614‐2.614)	.522
Age, y (>50 vs≤50)	1.152 (0.782‐1.698)	.475	0.918 (0.617‐1.364)	.671
PVC (without vs with)	0.532 (0.362‐0.782)	**.001**	0.662 (0.449‐0.977)	**.038**
Degree of PVTT ( type Ⅲ vs type Ⅰ‐Ⅱ)	0.759 (0.496‐1.163)	.205	0.857 (0.557‐1.317)	.315
Edmondson stage (Ⅲ‐Ⅳ vs Ⅰ‐Ⅱ)	1.030 (0.695‐1.526)	.882	1.142 (0.765‐1.705)	.515
Tumor number ( multiple vs single)	0.857 (0.579‐1.270)	.442	0.793 (0.537‐1.170)	.242
Tumor encapsulation ( complete vs none)	0.722 (0.350‐1.490)	.378	0.976 (0.534‐1.787)	.938
Tumor size, cm ( >5 vs ≤5)	1.746 (1.040‐2.932)	**.035**	3.869 (1.927‐7.770)	**<.001**
AFP (ng/mL), (>400 vs ≤400)	1.064 (0.721‐1.571)	.754	1.311 (0.882‐1.950)	.181
Anti‐HCV(positive vs negative)	1.340 (0.675‐2.662)	.403	0.736 (0.323‐1.680)	.467
HBsAg ( positive vs negative)	0.743 (0.406‐1.360)	.336	0.742 (0.422‐1.303)	.299
HBV DNA, IU/mL (>10^4^ vs ≤10^4^)	1.539 (0.997‐2.374)	.051	1.809 (1.203‐2.721)	**.004**
ALT,U/L ( >75 vs≤75)	1.076 (0.638‐1.814)	.783	1.033 (0.605‐1.765)	.905
GGT,U/L ( >54 vs ≤54)	1.840 (1.059‐3.199)	**.031**	2.032 (1.109‐3.726)	**.022**
Albumin, g/dL ( >3.5 vs ≤3.5)	1.268 (0.639‐2.516)	.497	1.653 (0.724‐3.733)	.233
PLT, ×10^9^ (>100 vs ≤100)	1.136 (0.714‐1.806)	.591	0.969 (0.609‐1.541)	.894
PT, ss ( >13 vs ≤13)	1.268 (0.707‐2.275)	.425	1.797 (1.050‐3.070)	**.033**
Liver cirrhosis (Yes vs No)	1.416 (0.958‐2.092)	.081	1.510 (1.015‐2.247)	**.042**
Child‐Pugh class (B vs A)	0.918 (0.445‐1.893)	.816	0.607 (0.247‐1.493)	.277
Multivariate analysis				
PVC (without vs with)	0.532 (0.361‐0.784)	**.001**	0.591 (0.395‐0.883)	**.010**
Tumor size, cm (>5 vs ≤5)	1.701 (1.001‐2.890)	**.049**	3.555 (1.744‐7.245)	**<.001**
HBV DNA, IU/mL (>10^4^ vs ≤10^4^)	NA	NA	1.498 (0.981‐2.287)	.061
PT, s ( >13 vs ≤13)	NA	NA	1.456 (0.828‐2.563)	.192
GGT,U/L ( >54 vs ≤54)	1.596 (0.911‐2.796)	.103	1.487 (0.798‐2.773)	.212
Liver cirrhosis (Yes vs No)	NA	NA	1.325 (0.884‐1.985)	.172

Note

Significant *P*‐values are shown in bold.

Abbreviations: AFP, alpha‐fetoprotein; ALT, alanine aminotransferase; CI, confidence interval; GGT, gamma glutamyl transpeptidase; HBsAg, hepatitis B surface antigen; HCV, hepatitis C virus; HR, hazard ratio; NA, not applicable; OS, overall survival; PLT, platelet count; PVC, portal vein chemotherapy; PVTT, portal vein tumor thrombosis; PT, prothrombin time; TTR, time to recurrence.

### 3.5 Postoperative PVC prevents early recurrence in patients with HCC and PVTT

**Figure 5:**
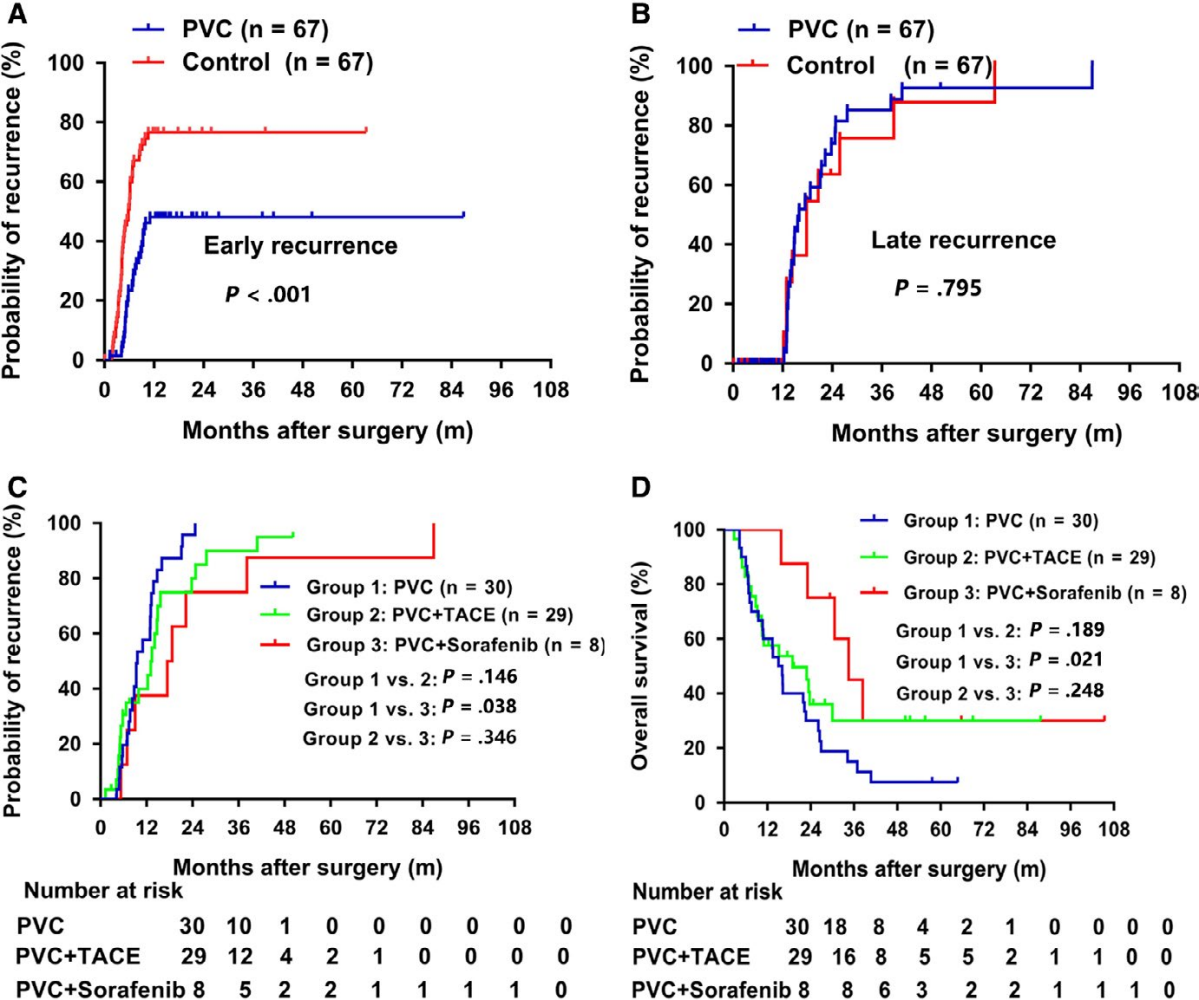
Effect of PVC and postoperative adjuvant sorafenib or TACE in preventing recurrence and prolonging survival. Kaplan‐Meier analysis of TTR for PVC treatment in early recurrence group (A) and late recurrence group (B). Postoperative PVC plus sorafenib could significantly prolong TTR (C) and OS (D) compared to PVC alone in HCC patients received PVC

To further define the role of PVC, we investigated its relation to postoperative tumor recurrence. At the time of last follow‐up, the cumulative recurrence rates were both 79.1% (53/67). However, the early recurrence (≤1 year) was significantly less frequent in PVC group (27/67) than those in the control group (43/67) (40.3% vs 64.2%, *P* = .006). Further Kaplan‐Meier analyses indicated that the probability of early recurrence in the PVC group was significantly lower than that of control group (*P* < .001) (Figure 5A), while no significant difference was found between the PVC group and the control group in regard to the probability of late recurrence (*P* = .795) (Figure 5B). Thus, PVC improves TTR primarily by reducing the probability of early recurrence after operation.

### 3.6 Combining postoperative PVC and other adjuvant treatments in patients with HCC and PVTT

Postoperative adjuvant TACE might effectively eliminate intrahepatic small residual tumors and strengthen the effectiveness of surgery;^23^, ^24^ 29 patients in PVC group and 35 patients in control group were treated with 1‐2 courses of adjuvant TACE treatment after operation. In recent years, sorafenib has been established as a new standard treatment option for advanced HCC;^8^, ^25^ eight patients in PVC group and four patients in control group were administered with postoperative sorafenib treatment. In addition, 30 patients in PVC group and 28 patients in control group received postoperative adjuvant treatments. There were no significant differences between PVC group and control group in postoperative treatment (*P* = .374). We further explore the effect of PVC combined with other adjuvant treatments, and found that postoperative PVC plus sorafenib significantly improved TTR and OS compared with PVC alone (TTR: 18.1 vs 9.6 months, *P* = .038; OS: 34.5 vs 15.6 months, *P* = .021), while there were no significant difference in TTR and OS between the PVC plus TACE group and the PVC group (TTR: 9.6 vs 13.4 months, *P* = .146; OS: 15.6 vs 19.0 months, *P* = .189) (Figure 5C). In addition, there were no significant differences in TTR or OS between the PVC plus sorafenib group and the PVC plus TACE group (TTR: 18.1 vs 13.4 months, *P* = .346; OS: 34.5 vs 19.0 months, *P* = .248), which might be due to the relatively small number of patients who received postoperative sorafenib (Figure 5D). We also compared the clinicopathological parameters among three groups and found that no parameters were statistically different between PVC group and PVC plus sorafenib group (Table S2). Therefore, we speculated that the differences in TTR and OS between PVC group and PVC plus sorafenib group were mainly determined by postoperative treatments.

## 4 DISCUSSION

Managing patients with HCC and PVTT is challenging,^4^, ^26^ but it may be possible to improve survival through surgical resection.^12^ Unfortunately, clinical outcomes are still dismal due to high rates of postoperative relapse and metastasis.^18^ In this study, we found that postoperative PVC could significantly prolong median TTR and OS intervals in HCC patients with PVTT, compared with untreated PSM controls, reducing the risk of recurrence and death by 48.3% and 39.7%, respectively. Our data have also shown that postoperative PVC significantly reduces the rate of early recurrence (≤1 year) in this setting, thereby improving OS.

There are clinical benefits to a sequential therapeutic approach such as this. During initial surgical procedures, primary tumors and portal vein thrombi are removed, relieving pressure in the portal vein. Intractable ascites and bleeding esophageal varices are thus prevented. Restoration of portal venous flow also improves liver function, increasing patient tolerance of chemotherapy; reducing tumor burden may increase the efficacy of postoperative multimodality treatments. PVC is a more targeted (regional) form of chemotherapy, enabling high local drug concentrations with minimal systemic side effects to eliminate residual tumors within the liver. The combination of surgical and PVC treatment stands to significantly improve clinical outcomes of patients with HCC and PVTT, having prolonged medians of TTR and OS in our patients by 5.6 and 6.5 months, respectively. Most importantly, PVC and sorafenib in combination further prolonged TTR and OS, compared with postoperative PVC alone, enhancing long‐term survival in some patients through multidisciplinary management. In conjunction with surgery, postoperative PVC plus sorafenib may be a promising therapeutic strategy under these circumstances, helping to reduce tumor recurrences and improve clinical outcomes.

To date, managing patients with HCC and PVTT has been complicated and controversial, with PVTT viewed as a relative or absolute contraindication to surgical resection. However, several reports have shown that hepatectomy with thrombectomy or en‐bloc resection may improve their survival.^27^, ^28^ As more studies accrue, especially in the Asia‐Pacific region, the safety and efficacy of performing surgical resection in some instances of PVTT is now validated.^28^ The median reported postoperative morbidity and in‐hospital mortality rates are 33% (range, 4‐50%) and 2.7% (range, 0.2‐11.5%), respectively. At our institute, 30‐day mortality after hepatectomy is only 1.7% (6/401). However, we reserve surgical intervention to patients with good general liver conditions, adequate liver functional reserves, absence of extrahepatic metastases, and tumor resectability. PVTT may culminate in broad hepatic and extrahepatic tumor dissemination, and even after resection, the postoperative recurrence rate is extremely high.^28^, ^29^ In a recent systematic review, the postoperative recurrence rate was as high as 80% (range, 28.5‐88%) in patients with macrovascular HCC invasion followed‐up for 25 months postoperatively.^7^ The cumulative recurrence rate was 79.1% for whole matched patients (n = 134). Thus, multidisciplinary management should be devised and implemented after surgery to reduce postoperative recurrences and tumor metastasis.

PVC has typically been used to prevent hepatic metastasis of colorectal or pancreatic cancer.^30^, ^31^ Few studies have addressed the clinical efficacy of PVC in managing patients with HCC and PVTT, although we have previously documented the potential clinical benefits of continuous PVC (ie, improved survival and fewer recurrences) in such patients.^13^, ^14^, ^15^ Liang et al have likewise determined that patients undergoing surgical resection, portal thrombectomy, and PVC for HCC with PVTT fare better in terms of median OS than those limited to resection and thrombectomy only (11.5 vs 6.2 months; *P* = .007).^11^ However, their small sample size brought conflicting conclusions, leaving the role of PVC for this purpose cloaked in controversy.^10^

PVC procedures customarily adhere to specific principles, one being that small HCC lesions are chiefly supplied by portal systems.^32^, ^33^ Previous studies have confirmed that tumors >10 mm are largely sustained by hepatic arteries, whereas those measuring 1‐5 mm are fed by hepatic and portal veins.^9^ According to experimental data, prophylactic portal vein chemotherapy serves to eliminate or prevent the seeding and neoproliferation of cancer cells.^34^ Our findings further support this premise, indicating that postoperative PVC reduces early recurrences (<1 year), ostensibly eradicating residual deposits that go undetected clinically. Notably, HCC patients with early recurrence had significantly worse survival rate.^35^ Hence, PVC administration after total removal of gross tumor and tumor thrombus is a valid means of reducing tumor recurrences and improving patient prognosis.

As for agents of preference in PVC, doxorubicin (60‐75 mg/m^2^) has shown modest efficacy and tolerability in patients with inoperable HCC, and recent studies have established that even sorafenib and doxorubicin in combination are tolerated by patients with advanced HCC.^36^ Although the survival benefit of doxorubicin is still in question, it is routinely and widely incorporated into chemotherapeutic regimens for HCC.^37^ Cisplatin and 5‐FU are commonly used to safely and effectively treat patients with unresectable and recurrent HCC tumors, delivered via hepatic artery infusion.^38^ In a cohort with multiple recurrences of HCC, Okuda K et al have reported a significantly higher 5‐year survival rate and effective responses through hepatic artery infusions of cisplatin and 5‐FU, compared with TACE and lipiodolization (45.7% vs 5.6% [*P* = .0274] and 71% vs 47.6% [*P* = .0171], respectively).^39^ Similarly, Itamoto et al have verified the efficacy of 5‐FU and cisplatin in seven patients with HCC and PVTT, recording a 33% tumor response rate and a MST of 7.5 months.^40^ Given these results, we used a combination of cisplatin, 5‐FU, and doxorubicin at relatively low doses for continuous (48‐hour) PVC administration in our patient population. This PVC regimen has significantly improved patient prognosis in the course of past studies and has performed even better during this investigation, prolonging OS in PSM analysis.^22^, ^41^

Postoperative multidisciplinary management has been advocated to reduce recurrences or metastasis of HCC in patients surgically treated for PVTT.^28^, ^42^ In some of our patients, PVC was also combined with other adjuvant treatments, such as TACE and sorafenib. By exploring these treatment subgroups, we found that postoperative PVC plus sorafenib (vs PVC alone) could significantly improve TTR and OS. Yet this was not true of PVC plus TACE, which proved similar to PVC alone in terms of TTR or OS. Although our available patients were few, a postoperative multidisciplinary protocol of surgical treatment and combination PVC/sorafenib may well contribute to long‐term survival of patients with advanced HCC and PVTT. A larger prospective study examining the feasibility and safety of this strategy is certainly warranted.

We additionally performed subgroup analysis of various factors that potentially impact patient prognosis in this setting. Those patients with type III PVTT, multiple tumors, or cirrhosis were likely to derive more clinical benefit than their counterparts, indicating that PVC could reduce the risks of recurrence and death in patients with unfavorable clinicopathological factors.^18^, ^23^ Furthermore, a high HBV DNA level adversely affected survival, likely due to impaired hepatic function. Consequently, use of antiviral treatment is advisable at levels >10^4^ IU/mL during PVC administration.^43^ However, interpretation of subgroup analysis should be cautious because of the relatively small number of patients in some subsets and substantial heterogeneity existing between different subpopulation besides the studied factors.

Our study had several clear limitations. Firstly, this was a retrospective review conducted at a single center and based on a limited number of patients. Although inherently subject to selection bias, PSM was used to offset related imbalances and verify that PVC could reduce early recurrence of HCC patients after operation. As another issue, patients in our PVC and control groups received other multimodality treatments in various dosages and courses. Still, no significant group‐wise differences in treatment methods existed, and this is a real‐world phenomenon, reflecting variability in individual therapeutic tolerances. Finally, in most of our study population, the development of HCC was HBV related. Whether the survival benefit of postoperative PVC applies in the absence of HBV has yet to be determined.

In conclusion, these study findings indicate that postoperative adjuvant PVC may significantly reduce early tumor recurrences and confer a survival advantage over surgical resection alone in patients with HCC and PVTT, particularly those with type III PVTT or multiple tumors. Furthermore, the addition of sorafenib may enhance postoperative PVC efficacy. A larger, prospective, and randomized controlled trial is needed to validate these results.

## CONFLICT OF INTEREST

The authors declare no conflict of interest.

## Data Availability

All data included in this study are available upon request by contact with the corresponding author.
