# Supplementary material for: Chemotherapeutic perfusion of portal vein after tumor thrombectomy and hepatectomy benefits patients with advanced hepatocellular carcinoma: A propensity score‐matched survival analysis
Source: Cancer Med. 2019 Sep 30;8(16):6933–44. doi: 10.1002/cam4.2556 (PMC6853833; doi:10.1002/cam4.2556)
Supplement: Supplementary file 1 [file CAM4-8-6933-s001.docx]

| Supplementary Table 1. Univariate and multivariate Cox regression analyses of clinicopathologic characteristics in HCC patients with PVTT before PSM. | | | | | |
| --- | --- | --- | --- | --- | --- |
| **Variables** | **TTR** | |  | **OS** | |
|  | HR (95% CI)  (95% CI) | P value |  | HR (95% CI)  (95% CI) | P value |
| **Univariate analysis** |  |  |  |  |  |
| Sex (male vs. female) | 1.082 (0.750-1.560) | 0.674 |  | 1.279 (0.827-1.978) | 0.268 |
| Age, y (>50 vs.≤50) | 1.073 (0.862-1.335) | 0.527 |  | 1.061 (0.878-1.343) | 0.624 |
| PVC (without vs. with) | 0.756 (0.561-1.018) | 0.066 |  | 0.901 (0.663-1.224) | 0.503 |
| Degree of PVTT ( type Ⅲ vs. type Ⅰ-Ⅱ) | 0.836 (0.598-1.170) | 0.296 |  | 1.046 (0.745-1.468) | 0.795 |
| Edmondson stage (Ⅲ-Ⅳ vs. Ⅰ-Ⅱ) | 1.200 (0.961-1.498) | 0.107 |  | 1.230 (0.967-1.566) | 0.092 |
| Tumor number ( multiple vs. single) | 1.035 (0.832-1.288) | 0.775 |  | 1.234 (0.974-1.565) | 0.082 |
| Tumor encapsulation ( complete vs. none） | 1.058 (0.771-1.452) | 0.727 |  | 0.929 (0.660-1.309) | 0.674 |
| Tumor size, cm ( >5 vs. ≤5) | 1.561 (1.227-1.985) | **< 0.001** |  | 2.445 (1.813-3.298) | **< 0.001** |
| AFP (ng/ml), (>400 vs. ≤400) | 1.164 (0.934-1.450) | 0.176 |  | 1.599 (1.254-2.038) | **< 0.001** |
| Anti-HCV (positive vs. negative) | 0.866 (0.555-1.351) | 0.525 |  | 0.811 (0.490-1.344) | 0.811 |
| HBsAg ( positive vs. negative) | 0.712 (0.634-1.238) | 0.184 |  | 0.759 (0.532-1.083) | 0.128 |
| HBV-DNA, IU/ml (>10^4^ vs. ≤10^4^) | 1.224 (0.957-1.565) | 0.107 |  | 1.526 (1.187-1.962) | **0.001** |
| ALT,U/L ( >75 vs.≤75) | 1.106 (0.754-1.621) | 0.608 |  | 0.925 (0.6708-1.406) | 0.714 |
| GGT,U/L ( >54 vs. ≤54) | 1.214 (0.941-1.566) | 0.136 |  | 1.573 (1.171-2.114) | **0.003** |
| Albumin, g/dl (>3.5 vs. ≤3.5) | 1.243 (0.838-1.845) | 0.280 |  | 0.982 (0.651-1.344) | 0.931 |
| PLT, ×10^9^ (>100 vs. ≤100) | 1.029 (0.779-1.360) | 0.839 |  | 0.858 (0.646-1.140) | 0.291 |
| PT, seconds (>13 vs. ≤13) | 1.066 (0.782-1.453) | 0.688 |  | 1.424 (1.048-1.935) | **0.024** |
| Liver cirrhosis (Yes vs. No ) | 1.347 (1.078-1.682) | **0.009** |  | 1.244 (0.979-1.581) | 0.073 |
| Child-Pugh class (B vs. A ) | 0.933(0.587-1.485) | 0.771 |  | 1.212 (0.768-1.910) | 0.409 |
| **Multivariate analysis** |  |  |  |  |  |
| Tumor size, cm ( >5 vs. ≤5) | 1.591 (1.250-2.026) | **<0.001** |  | 2.206 (1.626-2.994) | **<0.001** |
| AFP (ng/ml), (>400 vs. ≤400) | NA | NA |  | 1.307 (1.018-1.679) | **0.036** |
| HBV-DNA, IU/ml (>10^4^ vs. ≤10^4^) | NA | NA |  | 1.295(0.997-1.682) | 0.052 |
| GGT,U/L ( >54 vs. ≤54) | NA | NA |  | 1.235 (0.908-1.680) | 0.179 |
| PT, seconds (>13 s. ≤13) | NA | NA |  | 1.317 (0.965-1.798) | 0.083 |
| Liver cirrhosis (Yes vs. No ) | 1.381 (1.105-1.726) | **0.005** |  | NA | NA |
| Significant P-values are shown in bold.  TTR, time to recurrence; OS, overall survival; HR, hazard ratio; CI, confidence interval; PVC, portal vein chemotherapy; PVTT, portal vein tumor thrombosis; AFP, alpha-fetoprotein; HCV, hepatitis C virus; HBsAg, hepatitis B surface antigen; ALT, alanine aminotransferase; GGT, gamma glutamyl transpeptidase; PLT, platelet count; PT, prothrombin time; NA, not applicable. | | | | | |

Supplementary table 2. Comparisons of clinicopathological parameters among patients receiving PVC, PVC and TACE, PVC and Sorafenib.

| Variable | Treatment | | |
| --- | --- | --- | --- |
|  | **PVC** | **PVC+TACE** | **PVC+Sorafenib** |
| Sex |  |  |  |
| Female | 3 (10.0%) | 2 (6.9%) | 0 (0%) |
| Male | 27 (90.0%) | 27 (93.1%) | 8 (100.0%) |
| Age (y) |  |  |  |
| ≤50 | 21 (70.0%) | 12 (41.4 %)*^a^ | 6 (75.0%) |
| >50 | 9 (30.0%) | 17 (58.6%) | 2 (25.0%) |
| Degree of PVTT |  |  |  |
| Type Ⅰ-Ⅱ | 21 (70.0%) | 19 (62.1%) | 6 (75.0%) |
| Type Ⅲ | 9 (30.0%) | 10 (34.5%) | 2 (25.0%) |
| Edmondson stage |  |  |  |
| Ⅰ-Ⅱ | 12 (40.0%) | 15 (51.7%) | 2 (25.0%) |
| Ⅲ-Ⅳ | 18 (60.0%) | 14 (48.3%) | 6 (75.0%) |
| Tumor number |  |  |  |
| Single | 21 (70.0%) | 10 (34.5%)* | 3 (37.5%) |
| Multiple | 9 (30.0%) | 19 (65.5%) | 5 (67.5%) |
| Tumor encapsulation |  |  |  |
| None | 22 (73.3%) | 28 (96.6%)*^a^ | 8 (100.0%) |
| Complete | 8 (26.7%) | 1 (3.4%) | 0 (0%) |
| Tumor size (cm) |  |  |  |
| ≤5 | 19 (10.0%) | 6 (20.7%) | 3 (12.5%) |
| >5 | 11 (90.0%) | 23 (79.3%) | 5 (87.5%) |
| AFP (ng/ml) |  |  |  |
| ≤400 | 10 (33.3%) | 16 (55.2%) | 4 (50.0%) |
| >400 | 20 (66.7%) | 13 (44.8%) | 4 (50.0%) |
| Anti-HCV |  |  |  |
| Negative | 30 (100.0%) | 26 (89.7%) | 7 (87.5%) |
| Positive | 0 (0%) | 3 (10.3%) | 1 (12.5%) |
| HBsAg |  |  |  |
| Negative | 2 (6.7%) | 5(17.2%) | 0 (0%) |
| Positive | 28 (93.3%) | 24 (82.8%) | 8 (100.0%) |
| HBVDNA (IU/ml) |  |  |  |
| ≤10^4^ | 18 (60.0%) | 22 (75.9%) | 7 (87.5%) |
| >10^4^ | 12 (40.0%) | 7 (24.1%) | 1 (12.5%) |
| ALT(U/L) |  |  |  |
| ≤75 | 25 (83.3%) | 25 (86.2%) | 6 (75.0%) |
| >75 | 5 (16.7%) | 4 (13.8%) | 2 (25.0%) |
| GGT(U/L) |  |  |  |
| ≤54 | 4 (13.3%) | 7 (24.1%) | 1 (12.5%) |
| >54 | 26 (86.7%) | 22 (75.9%) | 7 (87.5%) |
| Albumin (g/dl) |  |  |  |
| ≤3.5 | 3 (10.0%) | 1 (3.4%) | 0 (0%) |
| >3.5 | 27 (90.0%) | 28 (96.6%) | 8 (100%) |
| PLT (×10^9^/L) |  |  |  |
| ≤100 | 7 (23.3%) | 5 (17.2%) | 2 (25.0%) |
| >100 | 23 (76.7%) | 24 (82.8%) | 6 (75.0%) |
| PT(second) |  |  |  |
| ≤13 | 27 (90.0%) | 25 (86.2%) | 7 (87.5%) |
| >13 | 3 (10.0%) | 4 (13.8%) | 1 (12.5%) |
| Liver cirrhosis |  |  |  |
| No | 10 (33.3%) | 14 (48.3%) | 5 (67.5%) |
| Yes | 20 (66.7%) | 15 (51.7%) | 3 (37.5%) |
| Child–Pugh class |  |  |  |
| A | 27 (90.0%) | 29 (100.0%) | 8 (100.0%) |
| B | 3 (10.0%) | 0 (0%) | 0 (0%) |

* P value was less than 0.05 compared with PVC group.

a. Fisher’s exact test.

PVC, portal vein chemotherapy; PVTT, portal vein tumor thrombosis; AFP, alpha-fetoprotein; HCV, hepatitis C virus; HBsAg, hepatitis B surface antigen; ALT, alanine aminotransferase; GGT, gamma glutamyl transpeptidase; PLT, platelet count; PT, Prothrombin time.
